# Occurrence of Asymptomatic Acute Neuromyelitis Optica Spectrum Disorder-Typical Brain Lesions during an Attack of Optic Neuritis or Myelitis

**DOI:** 10.1371/journal.pone.0167783

**Published:** 2016-12-09

**Authors:** Su-Hyun Kim, Jae-Won Hyun, AeRan Joung, Sang Hyun Lee, Ho Jin Kim

**Affiliations:** 1 Department of Neurology, Research Institute and Hospital of National Cancer Center, Go-Yang, Korea; 2 Department of Radiology, Research Institute and Hospital of National Cancer Center, Go-Yang, Korea; Medical University Vienna, Center for Brain Research, AUSTRIA

## Abstract

We aimed to investigate the frequency of asymptomatic acute brain MRI abnormalities accompanying optic neuritis (ON) or myelitis in neuromyelitis optica spectrum disorder (NMOSD) patients with aquaporin-4 antibodies (AQP4-Ab). We reviewed 324 brain MRI scans that were obtained during acute attacks of ON or myelitis, in 165 NMOSD patients with AQP4-Ab. We observed that acute asymptomatic NMOSD-typical brain lesions accompanied 27 (8%) acute attacks of ON or myelitis in 24 (15%) patients. The most common asymptomatic brain abnormalities included edematous corpus callosum lesions (n = 17), followed by lesions on the internal capsule and/or cerebral peduncle lesions (n = 9), periependymal surfaces of the fourth ventricle (n = 5), large deep white matter lesions (n = 4), periependymal cerebral lesions surrounding the lateral ventricles (n = 3), and hypothalamic lesions (n = 1). If asymptomatic NMOSD-typical brain abnormalities were considered as evidence for DIS, while also assuming that the AQP4-IgG status was unknown, the median time to diagnosis using the 2015 diagnosis criteria for NMOSD was shortened from 28 months to 6 months (p = 0.008). Asymptomatic acute NMOSD-typical brain lesions can be accompanied by an acute attack of ON or myelitis. Identifying these asymptomatic brain lesions may help facilitate earlier diagnosis of NMOSD.

## Introduction

Brain magnetic resonance imaging (MRI) abnormalities are frequently observed in patients with neuromyelitis optica spectrum disorder (NMOSD) [1–6], and some characteristic MRI abnormalities are incorporated into the recently proposed diagnostic criteria for NMOSD [7]. However, information on the clinical symptoms associated with these brain MRI abnormalities is still limited. We previously examined the clinical characteristics of 106 seropositive NMOSD patients, and found that brain MRI abnormalities (72%) were more frequent than clinical brain symptoms (51%) [8]. Thus, certain brain MRI abnormalities in NMOSD may develop without any significant brain symptoms; however, no data are available regarding the time of occurrence of such asymptomatic brain MRI abnormalities. Recent 1-year longitudinal study indicated that no new silent brain lesions developed between relapses in NMOSD patients [9]. Therefore, it is speculated that asymptomatic brain lesions develop simultaneously during attacks of myelitis or optic neuritis (ON). In fact, in cases with severe motor and sensory deficits or visual disturbance due to myelitis or ON, it is often difficult to determine whether the accompanying brain lesions are symptomatic.

Considering that NMOSD-typical brain MRI abnormalities have certain unique characteristics according to location and configuration, the presence of brain lesions in patients with the cardinal features of NMOSD (longitudinally extensive transverse myelitis [LETM], or ON) [7] might serve as evidence for the occurrence of dissemination in space (DIS), regardless of the clinical symptoms. However, due to the absence of obvious symptoms, asymptomatic brain lesions may be overlooked, unless clinicians pay attention to their possible presence. In the present study, we aimed to assess the frequency of asymptomatic acute brain MRI abnormalities accompanying ON or myelitis in NMOSD patients with antibodies to aquaporin-4 (termed AQP4-Ab).

## Materials and Methods

We enrolled consecutive NMOSD patients with AQP4-Ab who visited the multiple sclerosis clinic of the National Cancer Center, Korea, from May 2005 to January 2016. Of the 233 patients in our cohort, 11 patients were excluded due to the lack of brain MRI data. A total of 784 brain MRI scans were performed in 222 patients, of which 324 scans in 165 patients were performed during attacks of ON or myelitis. The anti-AQP4 antibodies were examined using ELISA [10] and a cell-based assay (CBA) with a commercial slide kit (Euroimmun, Luebeck, Germany) [11]. This study was approved by the NCC Institutional Review Board and written informed consent was obtained from all patients.

All MRI scans were performed using a 1.5-T or a 3.0-T machine. Brain scans included T2-weighted imaging, fluid-attenuated inversion recovery (FLAIR), gadolinium-enhanced T1-weighted imaging, and/or diffusion-weighted imaging. Brain MRI was performed within 2 weeks after an acute attack and prior to administration of steroids. Asymptomatic brain lesions were defined as the absence of clinically overt acute brain lesion-related symptoms. All evaluations were performed by two neurologists (S.H.K, and H.J.K.) and one neuroradiologist (S.H.L), and consensus in the results was achieved. Acute lesions on brain MRI were defined as newly developed T2-hyperintense lesions accompanied by one of the following: 1) increased diffusion-weighed signals, 2) gadolinium enhancement, or 3) decrease in size on a follow-up MRI [12]. Lesions were classified according to their location, as follows: corpus callosum lesions; corticospinal tract lesions (involving the internal capsule and cerebral peduncle); lesions on the periependymal surfaces of the fourth ventricle (brainstem/cerebellum); hypothalamic lesions; large, confluent deep white matter lesions; and periependymal lesions surrounding the lateral ventricles [1, 7]. We retrospectively analyzed the demographic and clinical findings, including age, gender, attack history, and dates of the MRI scans.

## Results

We identified a total of 1554 attacks in 222 NMOSD patients over a median disease duration of 9 years (range, 1–32 years). Of these patients, 95 (43%) had 173 symptomatic brain attacks. Of the 324 brain MRI scans that were performed during attacks of myelitis, ON, or area postrema syndrome in 165 patients, 27 MRIs (8%) from 24 patients (15%) were accompanied by acute asymptomatic NMOSD-typical brain lesions (LETM: n = 13, ON: n = 8, simultaneous LETM and ON: n = 5, and LETM and area postrema syndrome: n = 1). Of 24 patients, 22 (92%) were female. The median age at disease onset was 31 (range 6–49 years) and the median disease duration was 9 years (range 3–18 years). The median EDSS score was 3.75 (range 0–10). Most asymptomatic brain lesions occurred before immunosuppressive therapy and asymptomatic brain lesions were found after immunosuppressive treatment in 6 MRIs taken from 5 patients. Furthermore, acute asymptomatic brain lesions were detected during interferon treatment in 5 MRIs taken from 5 patients.

**Fig 1:**
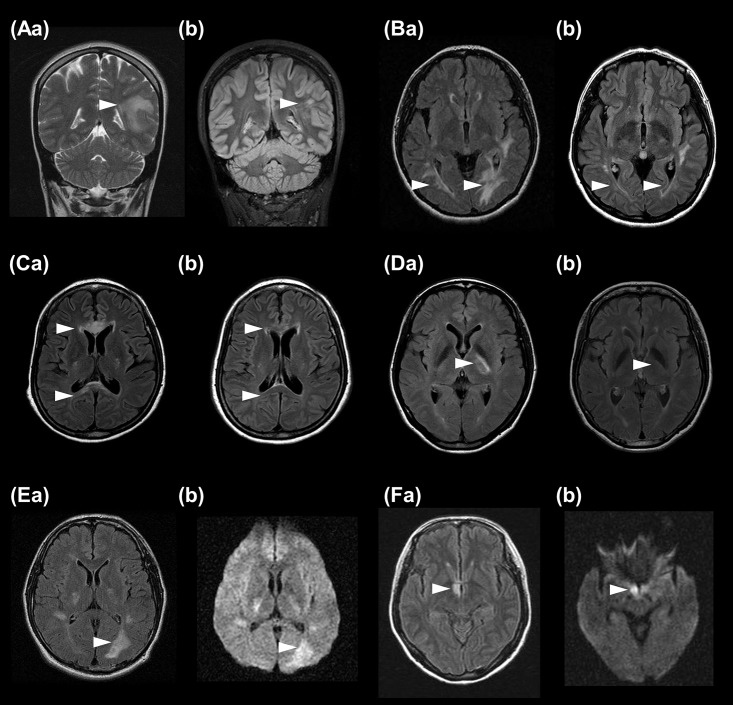
Asymptomatic acute brain lesions in AQP4-IgG seropositive patients with NMOSD during an acute attack of optic neuritis or myelitis. Examples of acute hyperintense lesions in the deep white matter (Aa), periependymal lining of the lateral ventricle (Ba), corpus callosum (Ca), and internal capsule (Da) on T2/fluid-attenuated inversion recovery (FLAIR) images during an acute attack of optic neuritis or myelitis. Note that lesions were markedly decreased in size or resolved on follow-up brain magnetic resonance images (Ab, Bb, Cb, Db). Example of a hyperintense lesion on FLAIR images with a high signal on diffusion-weighted images observed in the left periependymal lining of the lateral ventricle (Ea,Eb) and hypothalamus (Fa,Fb).

The most common asymptomatic brain abnormalities were corpus callosum lesions (n = 17), followed by lesions on the internal capsule and/or cerebral peduncle (n = 9), periependymal surfaces of the fourth ventricle (n = 5), large deep white matter lesions (n = 4), periependymal cerebral lesions surrounding the lateral ventricles (n = 3), and hypothalamic lesions (n = 1) (Fig 1). Among them, 19 lesions showed increased diffusion-weighted signals, 8 lesions had gadolinium enhancement, and 29 lesions revealed decrease in size at follow-up. Eleven (46%) of 24 patients exhibited multiple types of NMOSD-typical asymptomatic brain lesions. Additionally, 13 of 222 patients without any history of brain attacks showed NMOSD-typical brain lesions during the remission stage; however, it could not be determined when these lesions developed. Nevertheless, none of the patients exhibited newly developed brain lesions during the relapse-free period.

When asymptomatic NMOSD-typical brain abnormalities were considered as evidence for DIS, while assuming that the AQP4-IgG status was unknown, we found that the median time to diagnosis using the 2015 diagnostic criteria for NMOSD [7] was shortened from 28 months (range, 1–124 months) to 6 months (range, 0–96 months; p = 0.008) in the 24 patients. Forty-two (19%) of 222 patients had only recurrent myelitis or recurrent ON, with a median disease duration of 6 years (range, 1–32 years). If the AQP4-IgG status was in fact unknown for these patients, a diagnosis of NMOSD could not be made, as per the 2015 criteria [7]. Among these patients, 3 (7%) exhibited asymptomatic acute NMOSD-typical brain abnormalities on MRI during an attack of ON or myelitis.

## Discussion

In the present study, we found that asymptomatic acute NMOSD-typical brain lesions appeared in 8% of 324 attacks of myelitis or ON in 24 (15%) of 165 patients. Additionally, 13 patients did not exhibit any significant brain symptoms, but showed chronic NMOSD-typical lesions on MRI [12]. Thus, it appears that 37 (17%) of 222 patients may have simultaneously developed asymptomatic NMOSD-typical brain lesions during an attack of ON or myelitis.

Recently established 2015 diagnostic criteria facilitate the diagnosis of NMOSD, even in patients without AQP4-Ab [7]. These criteria require the presence of different core clinical characteristics to satisfy the detection of DIS in seronegative patients; however, asymptomatic brain lesions are not considered in the detection of the DIS component [7]. Nevertheless, with respect to neuroanatomical location, corpus callosum lesions, or periependymal cerebral lesions—which are not located in clinically eloquent regions—may be asymptomatic. Furthermore, certain lesions that were considered to be neurologically asymptomatic may in fact cause subtle neurologic manifestations that could be masked by more prominent symptoms in the context of an attack of ON or myelitis. A recent study reported that 28% of patients with NMOSD suffered from unrecognized and untreated depression which was also associated with neuropathic pain and fatigue [13]. The unrecognized depression and fatigue in NMOSD patients could also make us overlook subtle symptoms from brain lesions at least in some of our patients. In order to facilitate an earlier diagnosis of NMOSD, we question whether the distinction between symptomatic and asymptomatic brain lesions is necessary for establishing DIS. We previously reported that the early clinical events of NMOSD tend to recur in the same anatomical location within the central nervous system [14]. Hence, it might be difficult to satisfy two different core clinical characteristics during the early phase in some patients with NMOSD. In the present study, when asymptomatic brain lesions were considered as evidence for DIS, the time from disease onset to diagnosis was significantly shortened and 7% of the 42 patients with recurrent LETM or ON were diagnosed with NMOSD, even in the absence of AQP4-Ab data.

This study has limitations due to retrospective design and is based on data from a single center and the lack of serial assessment of MRI at regular intervals with standardized protocol. Some brain lesions may be considered asymptomatic simply due to the lack of any significant description of the brain symptoms in the patient’s medical history. Additionally, given that one-fourth of the acute brain lesions disappeared completely on follow-up MRI scans in patients with NMOSD [12], it is possible that we could have underestimated the frequency of asymptomatic acute brain lesions, as we did not assess the MRI scans performed during every attack of myelitis. Despite these limitations, our study showed that clinicians should consider the potential for accompanying brain lesions on MRI scans in patients presenting with ON or myelitis. Further prospective studies are needed to evaluate the frequency of asymptomatic acute brain lesions and to validate their impact on diagnosis and patient care in NMOSD.
